# Ketogenic Diet and Black Seed Oil (*Nigella sativa*) Combination Overcomes Doxorubicin Resistance in Breast Cancer: An In Vitro and In Vivo Study

**DOI:** 10.3390/nu18213610

**Published:** 2026-10-30

**Authors:** Rawan W. hadi, Majida Al wraikat, Moudi M. Alasmari, Doaa Anbarserry, Wamidh H. Talib, Bence Raposa

**Affiliations:** 1 Department of Clinical Nutrition and Dietetics, Applied Science Private University, Amman 11931, Jordan; rawanalyasari2001@gmail.com (R.W.h.);; 2 College of Medicine, King Saud bin Abdulaziz University for Health Sciences (KSAU-HS), Jeddah 21589, Saudi Arabia; 3 King Abdullah International Medical Research Centre (KAIMRC), Jeddah 21589, Saudi Arabia; 4 Ministry of National Guard Health Affairs (MNGHA), Jeddah 21589, Saudi Arabia; 5 Department of Clinical Pharmacology, Faculty of Medicine, King Abdulaziz University, Rabigh 21911, Saudi Arabia; 6 Faculty of Allied Medical Sciences, Applied Science Private University, Amman 11931, Jordan; 7 Institute of Basics of Health Sciences, Midwifery and Health Visiting, Faculty of Health Sciences, University of Pécs, 7621 Pécs, Hungary; bence.raposa@etk.pte.hu

**Keywords:** breast cancer, doxorubicin resistance, ketogenic diet, β-hydroxybutyrate, *Nigella sativa*, black seed oil, synergism, P-glycoprotein, VEGF, apoptosis

## Abstract

**Background:** This study evaluated the effects of black seed oil (BSO), a simulated ketogenic metabolic condition consisting of a low-glucose medium supplemented with 5 mM β-hydroxybutyrate (BHB), and their combination in EMT6/P and EMT6/DOX cells. The in vivo effects of a ketogenic diet, BSO, and their combination were also investigated in tumor-bearing mice. **Methods:** Cytotoxicity of black seed oil (BSO), a simulated ketogenic metabolic condition consisting of low-glucose medium supplemented with 5 mM β-hydroxybutyrate (BHB), their combination, and doxorubicin (DOX) was evaluated in EMT6/P and EMT6/DOX cells using the MTT assay. Combination index (***CI***) analysis was used to determine the interaction between BSO and the simulated ketogenic metabolic condition. Caspase-3 activity, vascular endothelial growth factor VEGF expression, and P-glycoprotein (P-gp) expression were evaluated in EMT6/DOX cells. In vivo, mice bearing EMT6/P and EMT6/DOX tumors received a ketogenic diet, BSO, their combination, DOX, and control treatment, and tumor growth, tumor weight, tumor-free rate, and serum biochemical parameters were assessed. **Results:** BSO half-maximal inhibitory concentration IC50 was 14.5-fold higher in EMT6/DOX than in EMT6/P, and DOX IC50 was 39.5-fold higher. The simulated ketogenic metabolic condition consisting of a low-glucose medium supplemented with 5 mM BHB + BSO combination gave a ***CI*** of 0.1244 in EMT6/P and 0.0239 in EMT6/DOX, indicating synergism in both lines. The combination produced the highest caspase-3 activity (1.54-fold vs. control), the lowest VEGF level (268.34 pg/mL vs. 953.34 pg/mL in control), and the lowest P-gp level (1.6 ng/mL vs. 3.7 ng/mL in control) among the treatments tested. In vivo, the combination reduced tumor volume by 87.15% in EMT6/P and 58.12% in EMT6/DOX, and gave a tumor-free rate of 66.6% in EMT6/P and 50% in EMT6/DOX. No consistent treatment-related change was observed in liver enzymes, renal markers, or lipid profile compared with healthy controls. **Conclusions:** The combination of a simulated ketogenic metabolic condition and BSO showed synergistic cytotoxic activity in vitro, while the ketogenic diet and BSO combination produced enhanced antitumor activity in vivo. These effects were associated with increased caspase-3 activity and reduced VEGF and P-gp expression in EMT6/DOX cells, without evidence of hepatic, renal, or metabolic toxicity. These findings support further investigation of this combination in doxorubicin-resistant breast cancer.

## 1. Introduction

Cancer remains a major health challenge in our era, becoming a leading cause of death globally, especially with the development of drug resistance [1,2]. In 2022, nearly 20 million new cancer cases were reported globally, resulting in approximately 10 million deaths attributed to the disease [1]. The cancers most commonly diagnosed include lung, breast, colorectal, prostate, and stomach cancers. Meanwhile, the primary causes of cancer-related deaths were lung, colorectal, liver, breast, and stomach cancers [1].

Breast cancer (BC) is the most frequently diagnosed cancer in women and a major contributor to cancer-related deaths among females worldwide [3]. According to the 2022 GLOBOCAN report, newly diagnosed cases of female breast cancer represented 11.6% of all cancer cases globally, making it the second leading cause of cancer mortality after lung cancer [1].

In clinical settings, key markers such as estrogen receptor (ER), progesterone receptor (PR), and human epidermal growth factor receptor 2 (HER2) are used for prognostic assessments, treatment choices, and eligibility for clinical trials. Based on the immunohistochemical evaluation of these markers, breast cancer is categorized into four subtypes: Luminal A, Luminal B, HER2-enriched, and triple-negative breast cancer (TNBC) [3].

Despite the progress made in chemotherapy, radiotherapy, immunotherapy, and targeted therapies, therapeutic resistance remains a leading cause of cancer-related deaths, contributing to up to 90% of cases [2]. Resistance to treatment can be categorized into two main types: intrinsic (or de novo) resistance and acquired resistance [3,4]. Intrinsic resistance refers to the inherent ability of certain cancers to resist specific cytotoxic drugs or combinations from the beginning, often due to pre-existing genetic changes or unique cellular characteristics. In contrast, acquired resistance develops during treatment as cancer cells evolve to withstand therapeutic pressures. Additionally, this form of resistance can result from the selection of pre-existing genetic mutations within the original tumors [5]. The mechanisms behind therapeutic resistance in cancer are complex and involve a range of factors, including genetic mutations, epigenetic changes, and cellular adaptability. Recent evidence highlights the abnormal expression and activity of different epigenetic modifiers in tumors, which result in unusual epigenetic changes that are strongly associated with both the aggressive nature of tumors and their resistance to treatment [6]. Epigenetic modifications, such as DNA methylation, histone alterations, and the regulation of non-coding RNAs, represent heritable changes in gene expression without alterations to the DNA sequence itself. These modifications are vital for regulating the expression of oncogenes and tumor suppressor genes and have been linked to the onset of cancer and the development of therapeutic resistance [6].

Given these limitations, there has been a growing interest in alternative and complementary therapeutic strategies, particularly those derived from natural sources. Phytochemicals and bioactive compounds extracted from medicinal plants have shown promising anticancer potential, often exhibiting selective cytotoxicity with fewer side effects compared to conventional chemotherapy [7]. Among these natural compounds, Thymoquinone (TQ), the primary bioactive component of Nigella sativa (black seed), stands out for its multi-targeted mechanisms of action against cancer. Recent preclinical research underscores TQ’s powerful anticancer properties [7], which include the induction of apoptosis [7] and regulation of autophagy [7]. Moreover, TQ has demonstrated significant promise in combination therapy, enhancing the effectiveness of conventional chemotherapeutic agents while reducing their associated toxicities [7].

*Nigella sativa* is also rich in a variety of bioactive compounds, including *thymol*, *carvacrol*, *p-cymene*, *α-pinene*, and *dithymoquinone*, and alkaloids like *nigellidine and nigellicine*, all of which play a role in its pharmacological effects [8]. The seeds are also highly nutritious, containing around 21% protein, 38–45% lipids, and 32% carbohydrates. They are a source of essential fatty acids, such as *linoleic and oleic acids*, in addition to vitamins and minerals like calcium, iron, zinc, and magnesium [8]. This rich combination of phytochemicals and nutrients enhances the therapeutic potential of *Nigella sativa* as both a nutraceutical and a complementary agent in cancer treatment [8].

Numerous studies have shown that *Nigella sativa* exhibits several mechanisms of action against chemotherapy-resistant cancer cells. It triggers apoptosis (programmed cell death) by activating specific stress signaling pathways, particularly the p38 MAPK pathway, which is crucial for inhibiting the growth of cancer cells and promoting the process of apoptosis [9]. Research has also indicated that *thymoquinone* can initiate apoptosis in chemotherapy-resistant cancer cells, irrespective of heightened levels of drug resistance proteins like O^6^-methylguanine-DNA methyltransferase MGMT or the activation of the extracellular signal-regulated kinase ERK pathway, both of which are significant factors in the failure of traditional chemotherapy [10]. Furthermore, *thymoquinone* aids in activating caspase enzymes, particularly Caspase-3, which facilitates the execution of apoptosis and helps to counteract drug resistance mechanisms, positioning it as a promising candidate for treating chemotherapy-resistant tumors [11].

A significant advancement in the understanding of cancer cell metabolism occurred in the 1920s, when Otto Warburg identified what is now referred to as the Warburg effect [12]. This finding demonstrated that cancer cells primarily depend on fermentation for energy, even in the presence of ample oxygen, due to impaired mitochondrial function [13]. This unique metabolic characteristic enables the rapid production of essential components required for cell division [14,15].

Driven by this insight, researchers have started investigating nutritional strategies, particularly ketogenic diets (KDs) and intermittent fasting, as complementary therapeutic options targeting the distinct metabolic needs of cancer cells [16].

A ketogenic approach, which consists of a high percentage of fats (80–90%), adequate protein, and significantly limited carbohydrates (2–8%), encourages a metabolic shift away from glucose-based energy production toward mitochondrial fat oxidation [14]. This shift shows potential in cancer treatment by attacking malignant cells while preserving the function of healthy tissues. Studies indicate that this dietary intervention lowers levels of insulin-like growth factor-1 (IGF-1) and boosts the production of ketone bodies, with animal model research suggesting that reduced carbohydrate intake can hinder cancer progression [14].

Recent studies suggest that ketogenic diets (KDs) may help combat drug resistance in cancer therapies through several molecular mechanisms [14]. By limiting glucose intake, this dietary strategy could potentially make resistant cancer cells more sensitive to chemotherapy [14]. Furthermore, the diet appears to inhibit the PI3K/Akt/mTOR pathway, which could improve the efficacy of targeted therapies [14]. Additionally, by reducing insulin and IGF-1 signaling, the diet may disrupt survival pathways that often drive treatment resistance [14].

The use of the ketogenic diet in cancer therapy was reported in different studies [14]. However, studies are lacking to evaluate the anticancer activity of KD combined with *Nigella sativa* oil to overcome doxorubicin resistance in breast cancer. This study tested increasing concentrations of black seed oil with fixed ketogenic conditions (low glucose + low BHB). The possible synergistic effect of this combination may improve the anticancer activity of KD and reduce its side effects.

## 2. Materials and Methods

### 2.1. Cell Lines and Culture Conditions

This study used two mouse mammary cell lines: the parent cell line (EMT6/P) and the doxorubicin-resistant cell line (EMT6/DOX), both obtained from the European Collection of Cell Cultures in Salisbury, UK. The cell lines were cultured using minimum essential medium that contained 10% fetal calf serum, 1% L-glutamine, 0.1% gentamicin, and 1% penicillin–streptomycin solution. The cells were incubated at 37 °C with 5% carbon dioxide and 95% humidity.

### 2.2. Antiproliferative (MTT) Assay

Mouse mammary cell lines (EMT6/P and EMT6/DOX) were cultured overnight. The actively growing cells were then harvested and assessed for viability using the trypan blue exclusion method. After cell counting, cells were seeded into 96-well tissue culture flat-bottom plates at a density of 10,000 cells per well and incubated for 24 h. The cells were then divided into the following treatment groups: untreated control, *Nigella sativa* oil (BSO) alone, simulated ketogenic metabolic condition alone, BSO combined with the simulated ketogenic metabolic condition, and doxorubicin (DOX) as a reference chemotherapeutic treatment. BSO, purchased from the Kingdom of Saffron shop (a local shop in Amman, Jordan), was tested at concentrations ranging from 0.78 to 100 μg/mL. The simulated ketogenic metabolic condition consisted of a low-glucose medium supplemented with 5 mM β-hydroxybutyrate (BHB), obtained from GENOCHEM WORLD, Bonrepòs i Mirambell, Valencia, Spain. For combination experiments, BSO was administered concurrently with the simulated ketogenic metabolic condition. All treatments were maintained for 48 h. Cell viability was subsequently evaluated using the MTT assay, and the resulting color development was measured using a microplate reader (BioTek, Winooski, VT, USA) at a wavelength of 555 nm. The percentage of cell survival for each treatment was calculated relative to untreated control cells. To assess the sensitivity of the parental and doxorubicin-resistant cell lines to the standard chemotherapeutic agent, increasing concentrations of DOX (0.78–100 μM; Ebewe Pharma, Unterach am Attersee, Austria) were also applied. The half-maximal inhibitory concentration (IC50) values were determined for both cell lines, and the resistance index (RF) was calculated according to the following formula: RF = IC50 of resistant cells/IC50 of parental cells. Resistance fold (RF) was calculated as the ratio of the IC50 value in EMT6/DOX cells to that in EMT6/P cells. The same terminology (RF) is used consistently throughout the manuscript.

### 2.3. Combination Index Calculation

To evaluate the interaction between black seed oil (BSO) and the simulated ketogenic metabolic condition, combination index (***CI***) values were determined using the Chou-Talalay method based on the median-effect principle. Briefly, EMT6/P and EMT6/DOX cells were exposed to serial dilutions of BSO (0.78 to 100 µg/mL) either alone in standard culture medium and in combination with a fixed simulated ketogenic metabolic background (low-glucose medium supplemented with 5 mM β-hydroxybutyrate, BHB). Cell viability was assessed via MTT assay after 48 h of exposure, and ***CI*** values were calculated to categorize interactions as synergistic (***CI*** < 0.9), additive (***CI*** = 0.9–1.1), or antagonistic (***CI*** > 1.1) in this study. And interpreted as described in the equation:

$$CI=\left(D\right)1/\left(Dx\right)1+\left(D\right)2/\left(Dx\right)2+\alpha \left(D\right)1\left(D\right)2/\left(Dx\right)1\left(Dx\right)2$$

where

(Dx)1: The dose of *Nigella sativa* oil required to produce 50% cell kill when used alone.
(D)1: Dose of *Nigella sativa* oil required to produce 50% cell kill in combination with the simulated ketogenic metabolic condition.
(Dx)2: Level/intensity of the simulated ketogenic metabolic condition required to produce 50% cell kill alone.
(D)2: Level/intensity of the simulated ketogenic metabolic condition required to produce 50% cell kill in combination with *Nigella sativa* oil.
α = 0 for mutually exclusive mechanisms of action, and α = 1 for a mutually non-exclusive mechanism

### 2.4. Measuring Apoptosis Induction in Cultured Cells

The ability of *Nigella sativa* oil and a simulated ketogenic metabolic condition consisting of low-glucose medium supplemented with 5 mM BHB, alone and in combination, to induce apoptosis in drug-resistant cells was assessed by evaluating caspase-3 activity. Freshly growing cells EMT6/DOX were treated for 48 h with *Nigella sativa* oil, a simulated ketogenic metabolic condition consisting of low-glucose medium supplemented with 5 mM BHB, and their combination, close to the IC50 value for each cell line. Doxorubicin was used as a positive control. After treatment, cells were harvested, washed, and lysed using lysis buffer. Caspase-3 activity for different treatments was measured using instructions in the standard kit (caspase-3assay kit, Abcam, Cambridge, MA, USA).

### 2.5. Determination of Vascular Endothelial Growth Factor (VEGF) Expression

Drug-resistant cell lines were cultured for 24 h, then harvested using trypsin-EDTA treatment. Cells were suspended in culture media and assessed for their viability using the trypan blue dye exclusion method. Cells were seeded at a concentration of 1.3 × 10^6^ cells/10 mL media and incubated for 24 h in small tissue culture flasks (five flasks per cell line). Then, culture media were removed and replaced with fresh media containing *Nigella sativa* oil, a simulated ketogenic metabolic condition consisting of low-glucose medium supplemented with 5 mM BHB, and their combination; the concentrations were determined based on IC50 values. Cells treated with doxorubicin were used as positive controls, and untreated cells were used as negative controls. Cells were incubated for 48 h, then the culture media were removed and the cells were harvested. Mouse vascular endothelial growth factor (VEGF) ELISA kit (catalogue # RAB0510; Sigma-Aldrich, St. Louis, MO, USA) was used to measure the expression of Vascular Endothelial Growth Factor (VEGF) in treated cells.

### 2.6. Detection of P-Glycoprotein (P-gp) Expression by ELISA

To assess drug resistance to EMT6/doxorubicin, P-glycoprotein (P-gp) expression was quantified using an ELISA-based method. Cells were seeded and grown until 50–60% confluence, after which fresh culture medium containing the designated concentrations of *Nigella sativa* (black seed) oil, a simulated ketogenic metabolic condition consisting of low-glucose medium supplemented with 5 mM BHB, and their combination were applied. Following a 48 h intervention, cells from each treatment group were collected and lysed to obtain protein extracts. Cell lysates were centrifuged at 12,000 rpm for 15–20 min to remove debris, and 500 μL of the supernatant was transferred into antibody-coated 96-well plates.

The ELISA assay was performed according to the manufacturer’s instructions. Absorbance at 450 nm was measured using a microplate reader, and P-gp concentrations were determined using a standard curve generated from the provided protein standards. All experiments were performed in triplicate for each treatment concentration.

### 2.7. Mice

In this study, 60 Balb/C female mice were used. Mice were 4–6 weeks old and weighed 21–25 g/mouse. All animal experiments were approved by the Research and Ethics Committee at the Faculty of Allied Sciences, Applied Science Private University. The animals were housed in appropriate cages with wood-shaving bedding and had free access to food and water. The housing environment was maintained under controlled conditions, including a temperature of approximately 25 °C, relative humidity of 50–60%, adequate ventilation, and a 12 h light/dark cycle. No additional structural environmental enrichment (e.g., shelters, tunnels, or toys) was specifically provided.

### 2.8. Antitumor Activity on Experimental Animals

Actively proliferating breast cancer cells (EMT6/P and EMT6/DOX) were collected through trypsinization, and cell viability was evaluated using the trypan blue exclusion method. Tumor induction was carried out by injecting 100,000 cells (in 0.1 mL) into the abdominal region of each female Balb/C mouse. The injected cancer cells were allowed to grow for 10 days to establish new tumors. Tumor dimensions were measured using a digital caliper, and tumor volumes were calculated using the formula: (A × B^2^ × 0.5), where A represents the length of the longest side of the tumor, and B is the length of the perpendicular side. After tumor development, mice were randomized so that the average tumor volume in all groups closely matched. Very small and very large tumors were excluded. The tumor-bearing mice were categorized as follows:

EMT6/P group: This group includes mice inoculated with EMT6/P (the parent breast cancer cell line), and treatments were initiated 10 days post-tumor inoculation. Thirty tumor-bearing mice were assigned to this group, divided into five sub-groups (*n* = 6 for each sub-group). Sub-group 1 served as the negative control, receiving daily oral intake of 0.1 mL olive oil. Sub-group 2 was given 120,000 mg/kg/day of a ketogenic diet (KetoCal^®^; Nutricia North America, Gaithersburg, MD), which provides complete nutrition in a 4:1 ratio of fats to carbohydrates plus protein (72% fat, 15% protein, and 3% carbohydrate). For diet preparation, KetoCal^®^ was mixed with water at a 2:1 ratio to prepare a pellet and provided to the animals in feed form daily. Sub-group 3 received a daily oral dose of *Nigella sativa* oil (5 mg/kg/day) by gavage. Sub-group 4 was treated with a combination of the ketogenic diet (like sub-group 2) and *Nigella sativa* oil (5 mg/kg/day) by gavage. Sub-group 5 was administered doxorubicin through intraperitoneal injection at 6 mg/kg/day.

EMT6/DOX group: This group comprises mice inoculated with EMT6/DOX (the doxorubicin-resistant breast cancer cell line), and treatments were commenced 10 days after tumor inoculation. Thirty tumor-bearing mice were included in this group, divided into five sub-groups (*n* = 6 for each sub-group). Sub-group 1 was the negative control and was treated as described earlier. Sub-group 2 received daily intraperitoneal injections of doxorubicin (6 mg/kg/day). Sub-group 3 was placed on a ketogenic diet (as described above). Sub-group 4 was administered *Nigella sativa* oil (5 mg/kg/day). Finally, sub-group 5 received a combination treatment of *Nigella sativa* oil (5 mg/kg/day) and the ketogenic diet (as in sub-group 3).

All treatments were continued for 10 days. On day 10, tumor volumes were measured, mice were sacrificed, and dissected tumors were stored in 10% formalin. Mice without detectable tumors at the inoculation site indicated successful treatment.

### 2.9. Blood Sampling Timeline

Blood samples were collected at two time points. The first blood sample was collected after tumor establishment and immediately before initiation of the dietary and treatment interventions, providing the baseline measurement for the treatment period. A second blood sample was collected at the end of the 10-day treatment period, immediately before euthanasia. Serum was separated and used for the assessment of the biochemical parameters described below.

### 2.10. Liver Function Assessment

Liver functions were assessed by measuring key biochemical markers, including alanine aminotransferase (ALT) and aspartate aminotransferase (AST). These parameters are essential for evaluating hepatic integrity and detecting any potential liver dysfunction.

### 2.11. Kidney Function Assessment

Kidney function was evaluated through the analysis of creatinine and uric acid. These indicators provide comprehensive insight into renal performance and fluid-electrolyte balance.

### 2.12. Blood Glucose and Beta-Hydroxybutyrate (β-OHB) Measurement

Blood glucose levels and beta-hydroxybutyrate (β-OHB) were measured to assess glycemic control and ketone body metabolism. These parameters are important for understanding changes in energy metabolism under experimental conditions.

### 2.13. Lipid Profile Analysis

The lipid profile was analyzed to evaluate lipid metabolism and potential cardiovascular risk. This included the measurement of triglycerides, high-density lipoprotein (HDL), and low-density lipoprotein (LDL), which are key indicators of dyslipidemia and cardiovascular health.

### 2.14. Statistical Analysis

Data were expressed as mean ± SEM. Normality of the data was evaluated using the Shapiro–Wilk test, and homogeneity of variances was evaluated using Levene’s test. The assumptions for parametric analysis were met, and one-way ANOVA, followed by Dunnett’s post hoc test for comparisons between each treatment group and the control, and Tukey’s post hoc test for comparisons between the combination group and the other treatment groups, was used to assess differences among treatment groups. Statistical significance was considered at a *p*-value < 0.05. Nonlinear regression analysis to determine the IC50 values of treatment for various cell lines was conducted using the Statistical Package for the Social Sciences, version 18 (SPSS Inc., Chicago, IL, USA).

## 3. Results

### 3.1. Cytotoxic Effects

The cytotoxic effects of 0.78–100 μg/mL black seed oil (BSO), a simulated ketogenic metabolic condition consisting of low-glucose medium supplemented with 5 mM BHB, their combination, and doxorubicin (0.78–100 µM) were evaluated in EMT6/P and EMT6/DOX breast cancer cells using the MTT assay.

**Figure 1:**
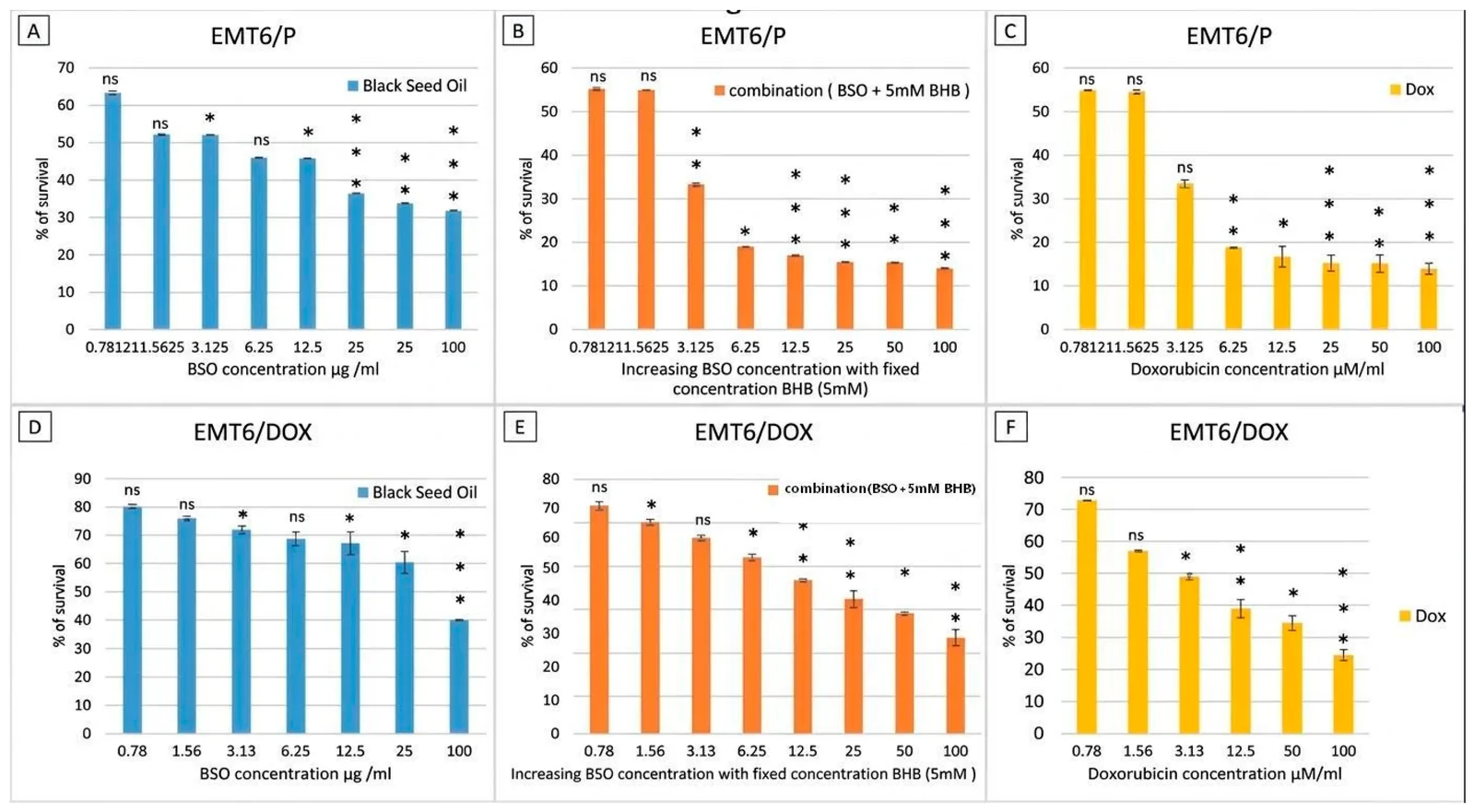
Cytotoxic effects of BSO, the simulated ketogenic metabolic condition, their combination, and doxorubicin on EMT6/P and EMT6/DOX cells. EMT6/P cells are shown in panels (**A**–**C**) and EMT6/DOX cells in panels (**D**–**F**). The simulated ketogenic metabolic condition consisted of a low-glucose medium supplemented with 5 mM BHB. Cell viability was assessed using the MTT assay after 48 h of treatment. Data are presented as Mean ± SD from independent experiments. Statistical analysis was performed using one-way ANOVA followed by Dunnett’s post hoc test to compare treated groups against the untreated control group (* *p* < 0.05,** *p* < 0.01, *** *p* < 0.001, and ns (not significant)).

The MTT assay demonstrated significant cytotoxic effects of BSO, a simulated ketogenic metabolic condition consisting of low-glucose medium supplemented with 5 mM BHB, their combination, and doxorubicin in both EMT6/P and EMT6/DOX cells (*p* < 0.0001) (Figure 1). In EMT6/P cells, cell survival ranged from 31.8 to 63.5% with BSO, 34.6–51.4% with a simulated ketogenic metabolic condition consisting of low-glucose medium supplemented with BHB, 31.1–44.8% with the combination, and 13.9–55.1% with doxorubicin (Figure 1A–C). In EMT6/DOX cells, survival ranged from 40.0 to 80.4%, 34.3–50.3%, 27.8–52.1%, and 22.6–73.1%, respectively (Figure 1D–F). The BSO IC50 increased from 6.27 ± 0.71 µg/mL in EMT6/P to 90.5 ± 6.8 µg/mL in EMT6/DOX, corresponding to a 14.5-fold resistance. Doxorubicin showed a 39.5-fold resistance in EMT6/DOX. Notably, the BSO+ simulated ketogenic metabolic condition consisting of a low-glucose medium supplemented with BHB produced strong synergism in EMT6/P (***CI*** = 0.1244) and very strong synergism in EMT6/DOX (***CI*** = 0.0239) (Table 1), indicating enhanced cytotoxic activity of the combination against both parental and resistant cells.

**Figure 2:**
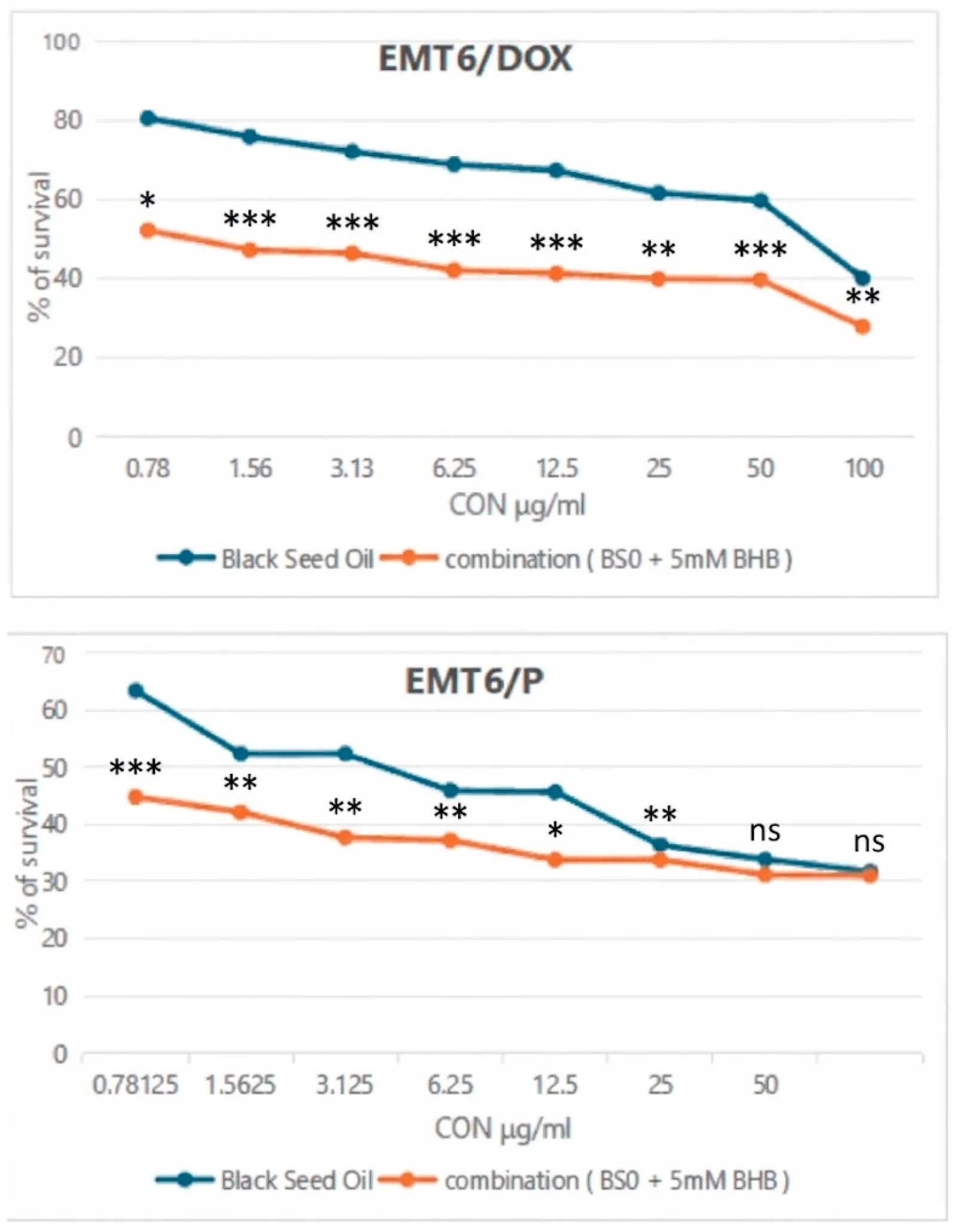
Dose–response profiles of EMT6/P and EMT6/DOX breast cancer cells treated with black seed oil (BSO) alone and in combination with a simulated ketogenic metabolic condition consisting of low-glucose medium supplemented with 5 mM BHB. Cell survival is expressed as a percentage across the tested BSO concentrations (0.78–100 µg/mL). Data represent the mean ± SD. Statistical significance was determined using one-way ANOVA followed by post hoc analysis, comparing the combination treatment to BSO alone at each concentration level: * *p* < 0.05, ** *p* < 0.01, *** *p* < 0.001, and ns (not significant).

The dose–response profiles of EMT6/P and EMT6/DOX cells following treatment with black seed oil (BSO) alone and in combination with a simulated ketogenic metabolic condition consisting of low-glucose medium supplemented with 5 mM BHB are presented in Figure 2. The combination treatment showed lower cell survival than BSO alone across most of the tested concentrations in both cell lines.

### 3.2. Effect of Treatments on Caspase-3 Levels in EMT6/DOX Cells

**Figure 3:**
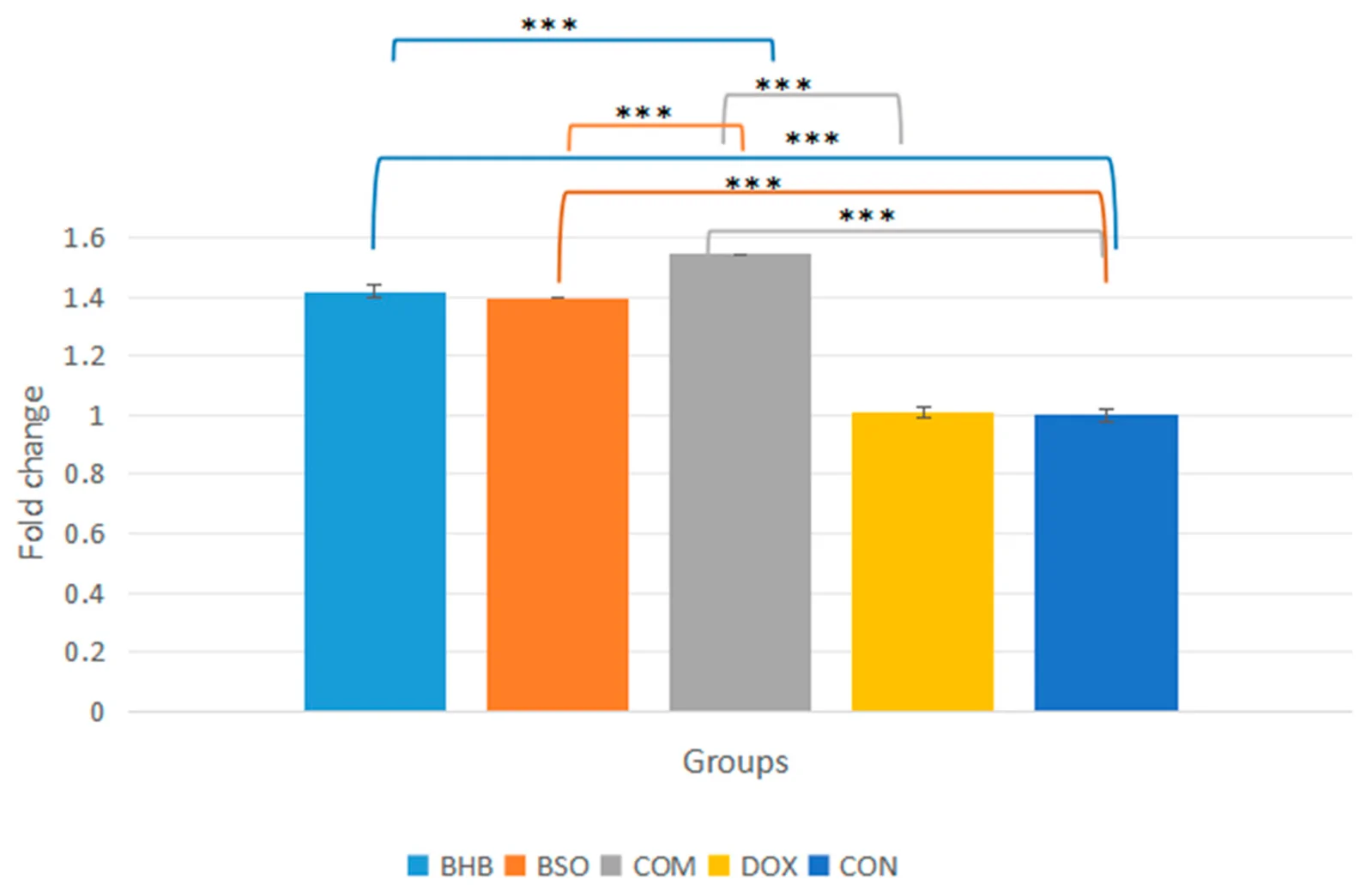
Activity of caspase-3 in EMT6/DOX cells following treatment with a simulated ketogenic metabolic condition consisting of low-glucose medium supplemented with 5 mM BHB, 45.5 μg/mL BSO, their combination (simulated ketogenic metabolic condition consisting of low-glucose medium supplemented with 5 mM BHB + 1.5 μg/mL BSO) and DOX (4.5 µM). Results are expressed as mean fold change ± SEM (*n* = 3). Significant differences are indicated as *** *p* < 0.001.

Treatment of EMT6/DOX cells with BSO (45.5 μg/mL), a simulated ketogenic metabolic condition consisting of low-glucose medium supplemented with 5 mM BHB and DOX (4.5 µM) for 48 h increased caspase-3 activity compared to the untreated control. BSO treatment caused a moderate increase in caspase-3 levels (~1.41-fold). A simulated ketogenic metabolic condition consisting of low-glucose medium supplemented with BHB treatment produced almost the same BSO effect (~1.39-fold). DOX treatment showed no significant effect on caspase-3 levels (~1.01-fold) compared with the control. The combined treatment (simulated ketogenic metabolic condition consisting of low-glucose medium supplemented with 5 mM BHB + 1.5 μg/mL BSO) resulted in the greatest induction of caspase-3 activity, reaching approximately 1.54-fold relative to control, and was significantly higher than either treatment alone, as well as the untreated control (Figure 3).

### 3.3. Effect of Treatments on VEGF Expression in EMT6/DOX Cells

**Figure 4:**
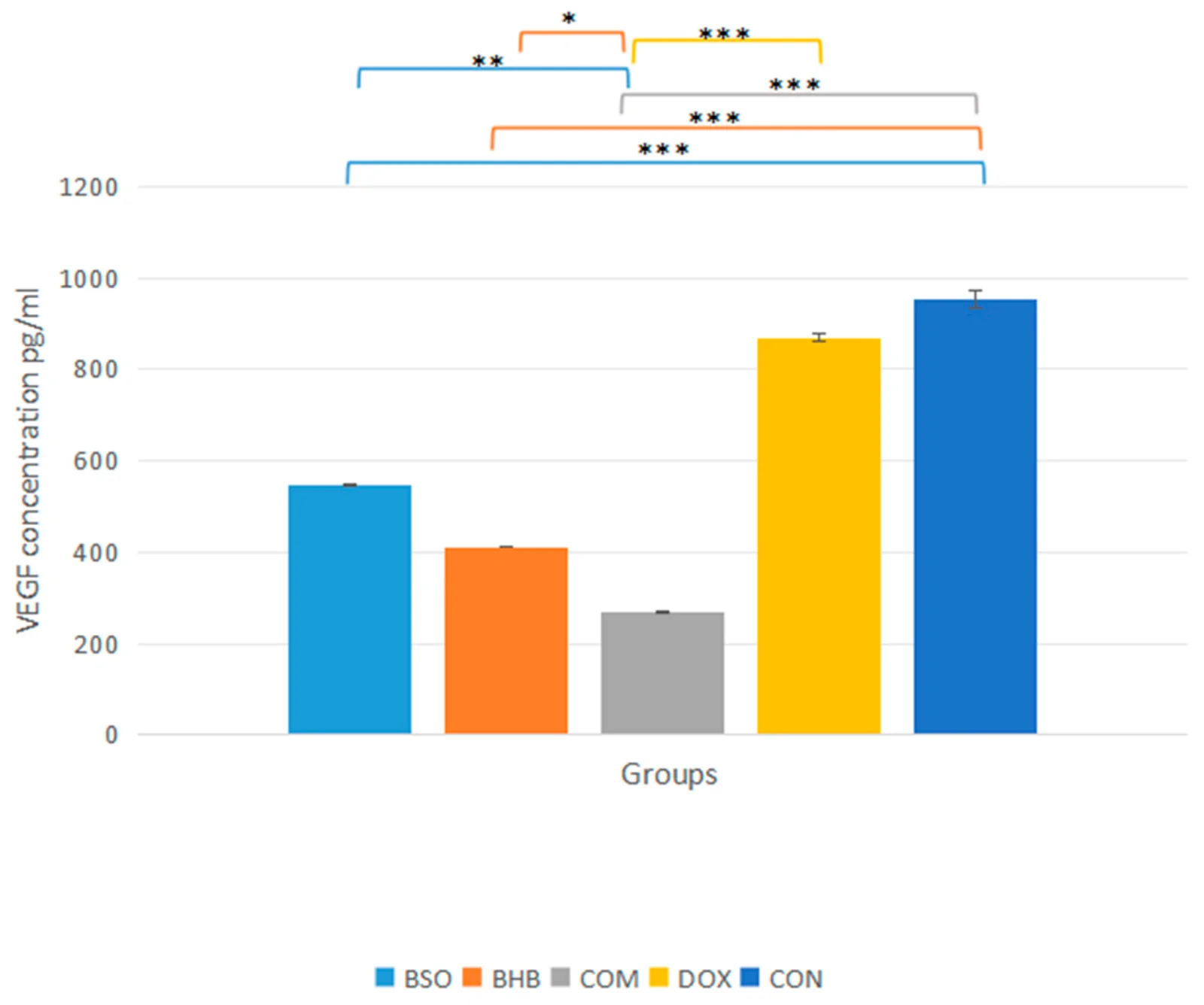
Effect of different treatments on VEGF expression by EMT6/DOX cells following treatment with a simulated ketogenic metabolic condition consisting of low-glucose medium supplemented with 5 mM BHB, 45.5 μg/mL BSO, their combination (simulated ketogenic metabolic condition consisting of low-glucose medium supplemented with 5 mM BHB + 1.5 μg/mL BSO) and DOX (4.5 μM). Results are expressed as mean VEGF concentration (pg/mL) ± SEM (*n* = 3). Significant differences are indicated as * *p* < 0.05, ** *p* < 0.01, and *** *p* < 0.001.

The anti-angiogenic potential of the different treatments was evaluated by measuring VEGF expression in cell culture. The untreated control group exhibited the highest VEGF levels (953.34 pg/mL). Single-agent treatment with BSO and BHB reduced VEGF levels to 545.13 pg/mL and 411.20 pg/mL, respectively, both representing statistically significant reductions compared to the untreated control (Figure 3). DOX treatment showed no significant reduction in VEGF levels 870.25 pg/mL compared to the control. The greatest reduction in VEGF levels was observed with the combination treatment (268.34 pg/mL), which was significantly lower than the BSO, DOX and control groups (Figure 4).

### 3.4. Effect of Treatments on P-Glycoprotein (P-gp) Expression in EMT6/DOX Cells

**Figure 5:**
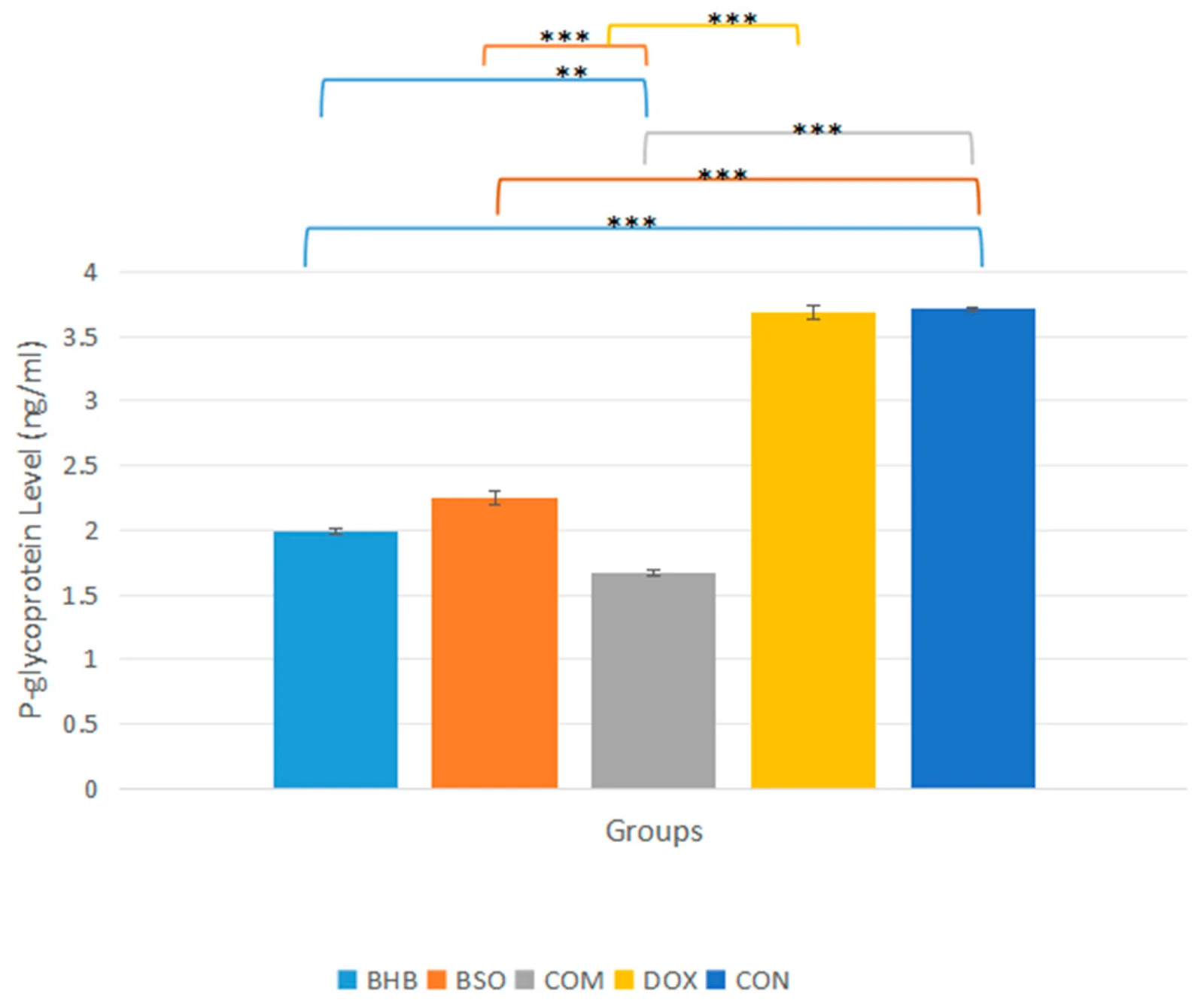
Effect of different treatments on P-gp expression by EMT6/DOX cells following treatment with a simulated ketogenic metabolic condition consisting of low-glucose medium supplemented with 5 mM BHB, 45.5 μg/mL BSO, their combination (simulated ketogenic metabolic condition consisting of low-glucose medium supplemented with 5 mM BHB + 1.5 μg/mL BSO) and DOX (4.5 µM). Results are expressed as mean p-gp concentration (ng/mL) ± SEM (*n* = 3). Significant differences are indicated as ** *p* < 0.01, and *** *p* < 0.001.

The untreated control group exhibited the highest P-gp levels (3.7 ng/mL). Single-agent treatment with BSO and BHB reduced P-gp levels to 2.2 ng/mL and 1.9 ng/mL, respectively, both representing statistically significant reductions compared to the untreated control. DOX treatment showed no significant reduction in P-gp levels 3.6 ng/mL compared to the control. The greatest reduction in P-gp levels was observed with the combination treatment (1.6 ng/mL), which was significantly lower than the BSO, DOX and control groups (Figure 5).

### 3.5. Anti-Tumor Effect Against Breast Cancer Implanted in Mice

A significant reduction in tumor size was observed in mice bearing EMT6/P tumors following treatment with the ketogenic diet (Keto), black seed oil (BSO), their combination (COM), and doxorubicin (DOX), compared with the untreated control (Table 2). The Keto and BSO treatments reduced tumor size by 64.87% and 64.24%, respectively, while the combination treatment achieved an even greater reduction of 87.15%. The greatest anti-tumor effect was observed in the doxorubicin-treated group, where the final tumor volume decreased to 0 mm^3^. In contrast, untreated control mice showed an 81.45% increase in tumor volume.

The reduction in tumor volume was accompanied by a decrease in final tumor weight. The lowest mean tumor weight was observed for the combination treatment (0.04 ± 0.03 g), compared to 0.28 ± 0.31 g for the ketogenic group and 0.09 ± 0.04 g for the BSO group. The highest rate of mice without tumors was observed in the doxorubicin group (100%), followed by the combination treatment group (66.6%), while Keto and BSO each achieved 33.3% without tumors. No remissions were observed in the control group.

In mice bearing EMT6/DOX tumors, all treatment groups showed a reduction in tumor volume compared to the untreated control group. Keto treatment resulted in a 49.30% reduction in tumor volume, while BSO and doxorubicin achieved reductions of 38.94% and 38.60%, respectively. The combination treatment showed the greatest reduction among the experimental treatments, at 58.12%. In contrast, the control group showed a slight increase in tumor volume of 1.81%.

The combination treatment also resulted in the lowest mean tumor weight (0.14 ± 0.08 g), compared to 0.18 ± 0.08 g for the Keto group, 0.33 ± 0.11 g for the BSO group, and 0.52 ± 0.46 g for the doxorubicin group.

The highest remission rate in mice was recorded in the combination group (50%), followed by the keto and doxorubicin groups (33.3%), and then the BSO group (16.6%), while no complete tumor remission was observed in the control group (Table 2).

**Figure 6:**
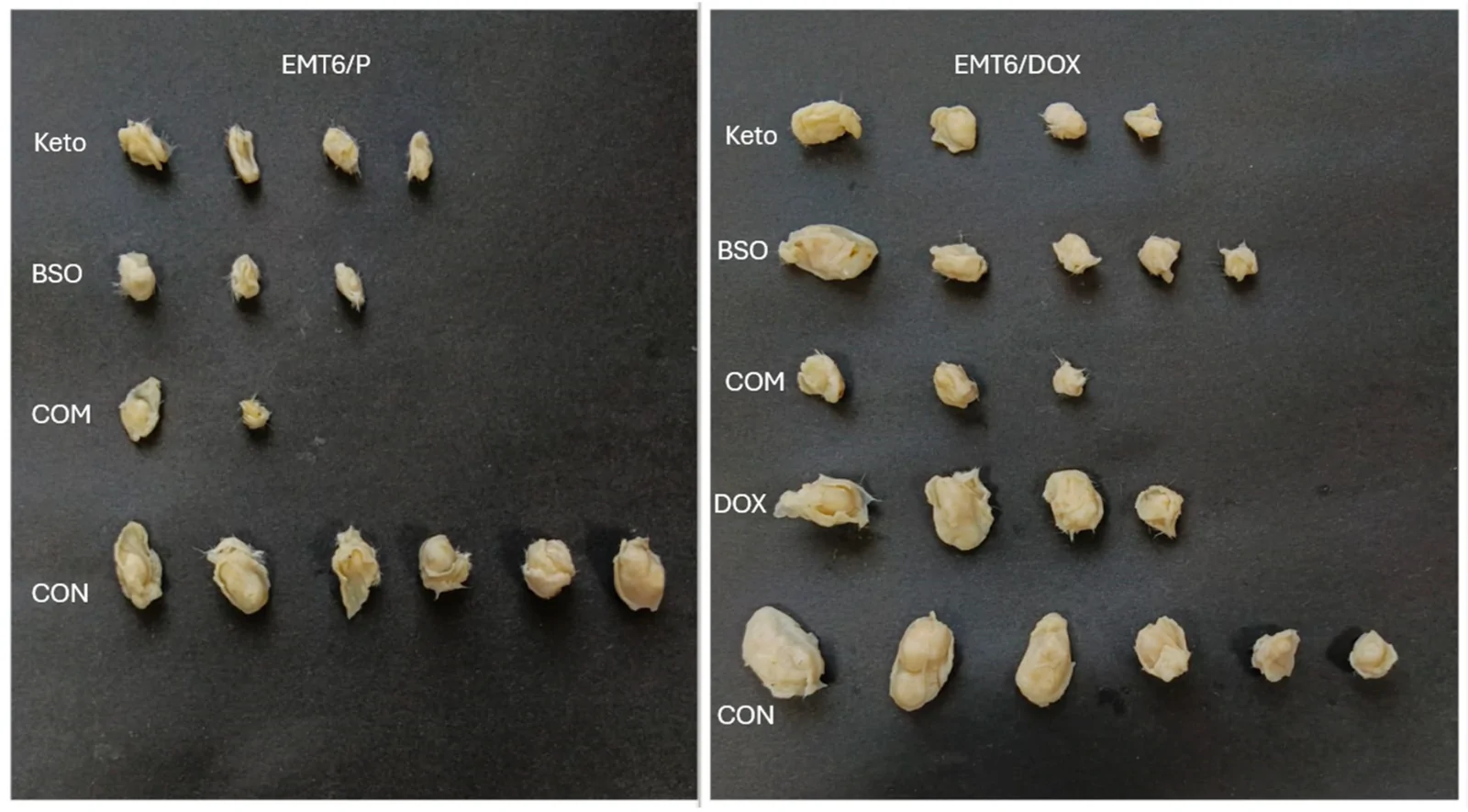
Comparison of final tumor sizes in mice treated with Keto, BSO, combination treatment, DOX and control (*n* = 6) for each group. Scale bar ≈10 mm. Tumors not shown indicate complete tumor regression. Quantitative tumor volume data are presented in Table 2.

Overall, the Keto + BSO combination demonstrated the strongest anti-tumor activity among all treatments, producing substantial tumor regression in both EMT6/P and EMT6/DOX models, with reductions of 87.15% and 58.12%, respectively, and a percentage of mice without tumor at the inoculation site of 66.6% and 50%. Final tumors collected on day 10 are shown in Figure 6 to illustrate differences in tumor size among the treatment groups.

### 3.6. Effect of Treatments on Serum Levels of AST, ALT, Creatinine, Blood Sugar, Uric Acid, Triglyceride, HDL and LDL for Each Group

Blood biochemical parameters were assessed to evaluate the potential metabolic, hepatotoxic, and renal effects of the different treatments. In both EMT6/P and EMT6/DOX treated mice, treatment-related differences were observed in blood glucose, uric acid, triglyceride, HDL, and LDL levels. Serum creatinine levels remained relatively stable between the treatment groups; ALT and AST levels also differed between the groups. However, no consistent treatment-related pattern was observed. Comparison with healthy mice supported the absence of a clear adverse effect of the treatments on liver and kidney function under experimental conditions Table 3.

## 4. Discussion

This study aimed to evaluate whether combining a simulated ketogenic metabolic condition consisting of low-glucose medium supplemented with BHB with black seed oil (BSO) could overcome doxorubicin resistance in breast cancer, both in vitro and in vivo. Overall, this finding suggests that the combination affects several mechanisms related to tumor growth and drug resistance, including cytotoxicity, apoptosis, angiogenesis, and P-gp activity. The effect of the combination was greater than that observed with either treatment alone, indicating a synergistic interaction. This effect was particularly evident in the doxorubicin-resistant cell line and tumors.

The EMT6/DOX cell line showed a 14.5-fold increase in the IC50 value of BSO and a 39.5-fold increase in the IC50 value of DOX compared to EMT6/P cells, indicating the successful establishment of the resistant model. The combination of ketogenic diet and BSO resulted in a strong synergistic effect in EMT6/P cells (***CI*** = 0.1244) and a very strong synergistic effect in EMT6/DOX cells (***CI*** = 0.0239). This synergy was stronger in the resistant strain, suggesting that this combination is not only more effective overall, but also interferes with the specific mechanisms responsible for resistance in EMT6/DOX cells. This is consistent with a dual mechanism of action: the induction of programmed cell death (associated with the thymoquinone compound) resulting from BSO [9,11], along with the metabolic stress imposed by the ketone-rich, glucose-poor environment on cells that depend on aerobic glycolysis for their growth (a phenomenon known as the Warburg effect).

The inhibitory effect observed with the simulated ketogenic metabolic condition should be interpreted in the context of both components of the treatment: reduced glucose availability and supplementation with BHB. Because the in vitro model used a low-glucose medium supplemented with 5 mM BHB, the reduction in cell viability cannot be attributed exclusively to BHB. Glucose restriction itself may impose metabolic stress on highly glycolytic cancer cells, whereas BHB may additionally influence cellular metabolism and signaling pathways [14,17]. Therefore, the observed cytotoxic effect likely reflects the combined influence of glucose restriction and BHB exposure rather than the independent effect of BHB alone. Future studies using matched glucose concentrations with and without BHB, together with appropriate metabolic controls, are required to distinguish the specific contribution of each component. Importantly, because the experimental condition combined glucose restriction with BHB supplementation, the present findings do not allow the independent contribution of glucose restriction to be separated from that of BHB. Therefore, the term “simulated ketogenic metabolic condition” is used to describe the in vitro treatment rather than implying that a complete ketogenic diet was reproduced in cell culture.

The combination produced the highest caspase-3 activity among all treatments (approximately 1.54-fold relative to the control), whereas DOX alone had almost no effect on the resistant cells (approximately 1.01-fold), consistent with the resistant phenotype of EMT6/DOX cells. The increase in caspase-3 with the combination was numerically modest, so programmed cell death is likely only a partial contributor to the overall cytotoxic effect, given how low the ***CI*** values were; there may also be other mechanisms, such as metabolic stress or modification of autophagy, but these were not measured here.

VEGF was lowest in the combined group (268.34 pg/mL vs. 953.34 pg/mL in untreated cells), consistent with an anti-angiogenic effect that would be expected to limit tumor blood supply in vivo. P-glycoprotein expression was also lowest in the compound group (1.6 ng/mL vs. 3.7 ng/mL in control), while DOX alone had little effect on P-gp (3.6 ng/mL). Given that P-gp is a key efflux transporter associated with doxorubicin resistance, this reduction offers a potential mechanism for the combination’s activity in resistant cells.

Mechanistic analyses of caspase-3 activity, VEGF expression, and P-glycoprotein expression were performed primarily in the EMT6/DOX cell line because the main objective of these analyses was to investigate potential mechanisms underlying the observed doxorubicin-resistant phenotype and the ability of the combination treatment to overcome resistance. Therefore, subsequent mechanistic experiments were focused on the resistant model to determine whether the combination treatment could enhance apoptosis, reduce angiogenic signaling, and modulate a major drug-efflux mechanism associated with doxorubicin resistance.

An additional limitation of the present study is that a standard pharmacological P-gp inhibitor was not included as a mechanistic comparator. Therefore, although the reduction in P-gp expression following the combination treatment supports a possible role of P-gp modulation in overcoming doxorubicin resistance, the present findings do not establish functional equivalence to direct pharmacological P-gp inhibition. Future studies should include a validated P-gp inhibitor as a comparator to further clarify the contribution of P-gp-mediated drug efflux to the observed reversal of doxorubicin resistance.

In EMT6/P tumors, DOX remained the most effective single treatment (100% reduction, 100% mice without tumor), an expected outcome for a chemosensitive model that validates the overall experimental design. In EMT6/DOX tumors, the combination reduced tumor volume by 58.12% and achieved a 50% cure rate, compared to a 38.6% reduction and a 33.3% cure rate for DOX alone. The reduction in tumor size in the DOX-resistant group after treatment with doxorubicin is possibly due to the reverse mutations that convert resistant cells into their sensitive original cells. These mutated cells can be eradicated by doxorubicin, which may explain the observed reduction in tumor size. In this resistant model, the group performed better than DOX on both measures. The tumor weight in the combined group was also lower than in the Keto or BSO groups alone in both models, consistent with a synergistic rather than additive effect in vivo, in line with in vitro ***CI*** values.

Levels of AST, ALT, creatinine, glucose, uric acid, triglycerides, HDL, and LDL showed no consistent treatment-related pattern compared to the healthy control group, indicating that neither the ketogenic diet nor BSO caused evident hepatic, renal, or metabolic toxicity at the dosages used. This supports the consideration of this combination as a potential adjuvant therapy rather than a substitute for standard chemotherapy.

Although the lipid profile was assessed as part of the metabolic safety evaluation, dedicated cardiovascular assessments or cardiac biomarkers were not included in the present study. Therefore, the available data do not allow definitive conclusions regarding the potential cardiovascular effects of the ketogenic diet or the combination treatment. Future studies should incorporate specific cardiovascular assessments to further evaluate the cardiovascular safety of prolonged ketogenic interventions.

Serum beta-hydroxybutyrate (β-OHB) levels did not rise significantly in the groups following the ketogenic diet after 10 days, despite the anti-tumor effects and biological mechanisms associated with this therapy. Several factors may explain this result.

The diet used in this study adhered to the classic 4:1 ketogenic ratio, representing the ratio of fat to the sum of protein and carbohydrates, which corresponds to approximately 90% of calories derived from fat. This ratio exceeds the minimum threshold required to induce ketosis in rodents; carbohydrate restriction alone does not lead to ketosis unless the diet is high in fat and low in protein [18]. Furthermore, diets with a fat content below approximately 85–90% have been shown to fail in raising blood β-OHB levels in mice [18]. Given that the formulation used here meets this threshold, insufficient fat content is an unlikely explanation for the absence of a significant rise in serum β-OHB; other factors discussed below are more likely responsible for this outcome.

One of the most probable explanations relates to the timing of blood sampling relative to feeding times; mice feed primarily during the dark phase, and blood ketone body levels decrease after food intake while rising during fasting [19]. If blood is not drawn from all groups at a standardized time following a specific fasting period, food intake immediately prior to sampling may mask the diet’s actual ketogenic effect; this is because postprandial insulin secretion inhibits hepatic ketogenesis, regardless of the diet’s overall composition.

Another possibility is that the tumor itself altered systemic ketone levels; certain tumors express ketone-degrading enzymes (such as BDH1 and OXCT1) and can take up circulating beta-hydroxybutyrate, oxidizing it for use as an energy source. In such cases, a ketogenic diet results in a smaller rise in blood ketone levels compared to tumor-free animals, as the tumor consumes these ketones [20]. Methodological factors must also be considered: beta-hydroxybutyrate levels may be underestimated due to delayed sample processing, hemolysis, or repeated freeze–thaw cycles. Furthermore, a single measurement at the 10-day mark might fail to capture an earlier peak followed by a phase of metabolic adaptation during which peripheral tissues increase their capacity to oxidize ketones over time, thereby lowering circulating beta-hydroxybutyrate levels even while ketogenesis continues. This finding should be reported as a study limitation. Future studies should standardize the timing of fasting and sampling and measure beta-hydroxybutyrate levels at multiple time points (e.g., days 0, 3, 7, and 10), given that the diet composition itself (4:1) fell within the expected ketogenic range.

These results are consistent with previous reports that a ketogenic diet can restore chemosensitivity when combined with other agents, including melatonin in cisplatin/vincristine-resistant breast carcinoma [21] and piperine combined with thymoquinone in breast carcinoma syngeneic grafts [22]. They also align with evidence that thymoquinone sensitizes resistant cancer cells to chemotherapy through caspase-3-dependent apoptosis [11] and modulation of ABC-transporter-mediated efflux [23]. This appears to be one of the first studies to combine a ketogenic/BHB condition with *Nigella sativa* oil in parallel parental and doxorubicin-resistant models, in vitro and in vivo.

Future studies should include an analysis of the immune balance between Type 1 and Type 2 helper T cells (Th1/Th2), as immunomodulation is a potential additional factor contributing to the observed synergistic anti-tumor effect, complementing previously reported findings regarding apoptosis, vascular endothelial growth factor (VEGF), and P-gp. The scope of statistical comparisons should also be expanded to include a direct three-way comparison between the keto, BSO, and doxorubicin treatment groups (in addition to the comparisons already made with the control group), so that the relative contribution of each factor individually to the effect of the combination therapy can be assessed directly, rather than being inferred only from comparisons with the control group.

## 5. Conclusions

The combination of a simulated ketogenic metabolic condition and black seed oil produced a synergistic cytotoxic effect against EMT6/P and EMT6/DOX breast cancer cells, while the ketogenic diet and BSO combination showed antitumor activity in vivo. In the resistant cell model, the in vitro combination was associated with increased caspase-3 activity and reduced VEGF and P-glycoprotein expression. In vivo, the combination produced greater tumor growth inhibition than either treatment alone and showed no significant difference in hepatic, renal, or metabolic toxicity under the experimental conditions compared with healthy mice. However, in vivo results showed no detection of ketone bodies in the blood of treated mice. This needs further investigation to address this limitation. Further studies are needed to have a better understanding of the mechanisms of action of this combination, including testing efflux functional assays, DOX retention studies, blood vessel formation, apoptosis signaling pathways, and histopathological evaluation.

## Figures and Tables

**Table 1:** IC50 values of BSO and doxorubicin and effective combination concentrations in EMT6/P and EMT6/DOX cells, with corresponding combination index (***CI***) values.

Cell Line	BSO (µg/mL)	Combination	Doxorubicin (µM)	Combination Index (*CI*)	Interpretation
EMT6/P	6.27 ± 0.71	<0.78 **	0.22 ± 0.1	0.1244	Very Strong Synergism
EMT6/DOX	90.5 ± 6.8	2.17 ± 0.17 **	8.7 ± 0.2	0.0239	Very Strong Synergism
Resistance Fold	14.5	2.78	39.5		

Interpretation of results: antagonism for ***CI*** value above 1.3; moderate antagonism for ***CI*** value of 1.1 to 1.3; additive interaction for ***CI*** value of 0.9 to 1.1; slight synergism for ***CI*** value of 0.8 to 0.9; moderate synergism for ***CI*** value of 0.6 to 0.8; synergism for ***CI*** value of 0.4 to 0.6; strong synergism for ***CI*** value of 0.2 to 0.4; and ***CI*** < 0.2 was interpreted as very strong synergism. Data are expressed as the mean ± SEM. Statistical significance was determined by comparing BSO in the combination with BSO alone. Significant differences are indicated as ** *p* < 0.01.

**Table 2:** Effects of Keto, BSO, and their combination on tumor size (mm^3^), and mice without tumor at the inoculation site.

EMT6/P					
Treatment	Initial tumor size (mm^3^)	Final tumor size (mm^3^)	Percentage change in tumor size	Average tumor weight (g)	Percentage of mice without tumor at the inoculation site
Keto	416.58 ± 17.4	146.34 ± 49.69	−64.87 *	0.28 ± 0.13	33.3%
BSO	394.71 ± 45.7	141.14 ± 54.21	−64.24 *	0.09 ± 0.04	33.3%
COM	423.22 ± 19.3	54.35 ± 34.44	−87.15 *#€	0.04 ± 0.03	66.6%
DOX	438.44 ± 29.8	0	−100 *	0	100%
CON	509.04 ± 27.9	923.70 ± 105.8	81.45	1.11 ± 0.06	0
EMT6/DOX					
Treatment	Initial tumor size (mm^3^)	Final tumor size (mm^3^)	Percentage change in tumor Size	Average tumor weight (g)	Percentage of mice without tumor at the inoculation site
Keto	307.01 ± 14.3	155.63 ± 49.9	−49.30 *	0.18 ± 0.08	33.3%
BSO	386.35 ± 79.3	235.89 ± 60.36	−38.94 *	0.33 ± 0.11	16.6%
COM	344.85 ± 13.8	144.42 ± 65.07	−58.12 *#€	0.14 ± 0.08	50%
DOX	501.21 ± 53.12	307.40 ± 110.89	−38.6 *	0.52 ± 0.46	33.3%
CON	369.9 ± 22.53	376.63 ± 78.61	1.81	0.37 ± 0.13	0

Data are expressed as mean ± SEM (*n* = 6). Statistical significance was defined as * *p* < 0.05 vs. control, # *p* < 0.05 vs. BSO-treated group, and € *p* < 0.05 vs. Keto-treated group. Mice without tumor in the inoculation site was defined as the absence of a detectable tumor at the inoculation site following 10 days of treatment period.

**Table 3:** Serum levels of AST, ALT, creatinine, blood sugar, uric acid, triglyceride, HDL and LDL across all experimental groups and the healthy group.

**EMT6/P**									
**Treatment**	**Blood Sugar mg/dL**	**Creatinine mg/dL**	**Uric Acid mg/dL**	**Triglyceride mg/dL**	**HDL mg/dL**	**LDL mg/dL**	**ALT U/L**	**AST** **U/L**	**Ketone Bodies**
BSO	168.5 ± 1.1	0.22 ± 0.01	2.51 ± 0.37	176.5 ± 16.5	30.5 ± 0.5	24	30.5 ± 1.22	157.2 ± 2.1	N
Keto	144.8 ± 2.1	0.19 ± 0.01	2.91 ± 0.31	211.5 ± 14.5	61.5 ± 1.5	39.5 ± 0.5	40.8 ± 1.6	213.6 ± 6.2	N
Com	153.7 ± 4.5	0.19 ± 0.003	6.64 ± 0.48	170 ± 10	55	27 ± 2	46 ± 3.6	189 ± 15.1	N
DOX	160.5 ± 3	0.15 ± 0.0088	2.3 ± 0.08	161.5 ± 13.5	25.6 ± 0.6	24 ± 3.4	35.9 ± 2.3	154.65 ± 3.45	N
CON	156.3 ± 4.15	0.14 ± 0.008	2.8 ± 0.41	144 ± 15	30.3 ± 4.7	34.6 ± 3.17	34.1 ± 3.1	254.1 ± 15.4	N
**EMT6/DOX**									
BSO	68.1	0.15 ± 0.0057	3.52 ± 0.719	158 ± 2	38.5 ± 0.40	42 ± 2.8	66.2 ± 2.75	277.2 ± 3.6	N
Keto	168.7 ± 5.2	0.10 ± 0.003	3.70 ± 0.70	142	55.3 ± 1.8	38.6 ± 4.3	92 ± 5.6	164 ± 13.4	N
COM	99 ± 20.2	0.11 ± 0.0033	3.04 ± 0.89	282 ± 31	50 ± 2.3	36 ± 5	36.9 ± 6.9	150.9 ± 11.6	N
DOX	130 ± 2	0.12 ± 0.012	2.06 ± 0.45	136.5 ± 6.5	28.6 ± 1.8	29.3 ± 2.33	45.15 ± 11.6	203.7 ± 11.6	N
CON	137.2 ± 4.9	0.15 ± 0.0033	4.9 ± 0.92	256.5 ± 20.5	28.6 ± 2.66	18.5 ± 1.5	43.15 ± 16.05	142.7 ± 9.7	N
Healthy	130.4 ± 7.2	0.19 ± 0.013	2.6 ± 0.66	155 ± 7	42.6 ± 3.4	39 ± 1	77.2 ± 2.2	175.4 ± 5.7	N

Values are expressed as mean ± SEM., not statistically significant (*p* ≥ 0.05), compared with the control group (CON) or healthy control group (HC), as indicated. N = not detected.

## Data Availability

The data presented in this study are available on request from the corresponding author.
